# Immuno-Histochemical Analysis of Rod and Cone Reaction to RPE65 Deficiency in the Inferior and Superior Canine Retina

**DOI:** 10.1371/journal.pone.0086304

**Published:** 2014-01-21

**Authors:** Daniela Klein, Alexandra Mendes-Madeira, Patrice Schlegel, Fabienne Rolling, Birgit Lorenz, Silke Haverkamp, Knut Stieger

**Affiliations:** 1 Department of Ophthalmology, Faculty of Medicine, Justus-Liebig-University Giessen, Giessen, Germany; 2 Translational Gene Therapy for Retinal and Neuromuscular Diseases, INSERM UMR 1089, Institut de Recherche Thérapeutique 1, Université de Nantes, Nantes, France; 3 Department of Computational Intelligence, Faculty of Mathematics and Computer Science, Philipps University Marburg, Marburg, Germany; 4 Max Planck Institute for Brain Research, Frankfurt am Main, Germany; University of Cologne, Germany

## Abstract

Mutations in the RPE65 gene are associated with autosomal recessive early onset severe retinal dystrophy. Morphological and functional studies indicate early and dramatic loss of rod photoreceptors and early loss of S-cone function, while L and M cones remain initially functional. The Swedish Briard dog is a naturally occurring animal model for this disease. Detailed information about rod and cone reaction to RPE65 deficiency in this model with regard to their location within the retina remains limited. The aim of this study was to analyze morphological parameters of cone and rod viability in young adult RPE65 deficient dogs in different parts of the retina in order to shed light on local disparities in this disease. In retinae of affected dogs, sprouting of rod bipolar cell dendrites and horizontal cell processes was dramatically increased in the inferior peripheral part of affected retinae, while central inferior and both superior parts did not display significantly increased sprouting. This observation was correlated with photoreceptor cell layer thickness. Interestingly, while L/M cone opsin expression was uniformly reduced both in the superior and inferior part of the retina, S-cone opsin expression loss was less severe in the inferior part of the retina. In summary, in retinae of young adult RPE65 deficient dogs, the degree of rod bipolar and horizontal cell sprouting as well as of S-cone opsin expression depends on the location. As the human retinal pigment epithelium (RPE) is pigmented similar to the RPE in the inferior part of the canine retina, and the kinetics of photoreceptor degeneration in humans seems to be similar to what has been observed in the inferior peripheral retina in dogs, this area should be studied in future gene therapy experiments in this model.

## Introduction

The retinal pigment epithelium protein of approximately 65 kDa in size (RPE65) plays a crucial role in the visual cycle, which restores the light sensitive chromophore 11-*cis* retinal out of the bleached all trans retinal [1], [2]. It is expressed in the RPE and in cones in the mammalian retina [3], [4], and in the zebrafish retina additionally in Müller cells [5]. To date, the RPE located RPE65 protein is supposed to be the exquisite source of 11-*cis* retinal for rods, while the supply for cones may be satisfied also by Müller cells and the cones themselves [6], [7]. Furthermore, an alternative source of isomerase activity in cones has been described [8].

Homozygous and compound heterozygous mutations in the RPE65 gene are associated with Leber’s congenital amaurosis (LCA) type 2 or with early onset severe retinal dystrophy (EOSRD), depending on the age of onset of severe visual impairment. Typical clinical signs include profound night blindness, reduced visual field, severely reduced or absent fundus autofluorescence (FAF) and absent rod ERG recordings [9]–[12]. Optical coherence tomography (OCT) scans reveal reduced outer nuclear layer (ONL) thickness early in life [13]. Chromatic pupillometry and chromatic sensitivity studies indicate an early loss of S-cone function, while L and M-cones remain initially functional [14].

Several natural occurring as well as genetically engineered animal models exist for RPE65 deficiency. The RPE65 knockout mouse was generated more than a decade ago, proving the function of the RPE65 protein in supplying 11cis retinal to the photoreceptors [15]. This mouse displays a severe phenotype. An R91W mutated mouse line was generated later, displaying a milder phenotype with early S-cone loss [16]. The rd12 mouse represents a naturally occurring mouse model with a moderate phenotype [17]. The naturally occurring canine model of RPE65 deficiency, the Swedish Briard dog, was discovered over 20 years ago, initially diagnosed with congenital stationary night blindness (CSNB) [18], [19]. However, it is clear now that the phenotype in the dog is progressive, representing a model for progressive retinal degeneration [20], [21].

All animal models have been employed in the development of a gene therapy approach for the treatment of RPE65 deficiency. Especially the canine model proved to be a valuable tool, representing a model of comparable size and with a similarly developed immune system as in humans [22]. Adeno-associated virus (AAV) vector mediated gene therapy in the superior, non-pigmented part of the retina demonstrated robust rescue of rods and cones, as shown by ERG recordings and ambulating an obstacle course, raising hope for a transfer of the results to the clinic [23]–[26].

Gene therapy studies in over 30 patients resulted in significant improvement in light sensitivity and increase in visual field, indicating a rescue effect for rods [27]–[34]. In contrast, visual acuity did not improve in treated patients, indicating a lack of rescue in central cones. Recent data in human patients obtained through OCT measurements indicate a gradual loss of ONL thickness even in treated areas, while rod photoreceptor function was improved [35]. The reason for the impact on rod, but not cone function remains unknown.

Similarly, the reason for the discrepancy in the rescue effect between dogs and humans is unknown. In this regard it should be noted that some differences exist in the superior part of the canine retina, which is normally targeted in preclinical trials, compared to the human retina besides obvious differences such as the lack of a macula region or a foveal pit. First, the superior part of the canine retina is attached to non-pigmented RPE and a unique layer is situated behind the RPE, the tapetum lucidum, which reflects incoming light and thus allows a second passage of photons through the outer segments enabling increased light captivity [36]. Second, the ONL thickness in the superior part of the canine retina seems to be preserved until late in the disease process and therefore, does not display similar degeneration kinetics compared to the situation in humans, where rapid ONL thinning is observed early [13], [20]. Interestingly, the inferior part of the canine retina, which is attached to pigmented RPE without a tapetum lucidum, has not yet been thoroughly studied with regard to degenerative processes.

In this study, we investigated for the first time the state of rod and cone viability in the inferior part of the canine retina and compared the data with the situation in the superior part. We analyzed sprouting of rod bipolar and horizontal cells and observed dramatic sprouting events in the inferior peripheral part of affected retinae while other parts of the retina remained silent. In contrast, while analyzing S- and L/M-cone opsin expression, we found a protection of S- cone opsin expression in the inferior part of the retina, but not in the superior part. This was not the case for L/M cone opsin. These observations indicate pronounced differences in rod and cone viability in the inferior part compared to the superior part of the canine retina, which may have important implications with regard to choosing the ideal treatment area in the canine retinae in order to model the human disease.

## Materials and Methods

### Ethics Statement

Local Institutional Animal Care and Use Committee (IACUC) approval was not necessary because the animals were euthanized at the end of original study (AAV.tetOn.RPE65 gene therapy in dogs [13], [20]) and eye cups were removed after euthanasia. The IACUC of the University of Nantes approved the protocol for the original study.

### Tissue Preparation

The dogs were housed at the Boisbonne Center (ONIRIS, Nantes-Atlantic College of Veterinary Medicine, Food Science and Engineering, Nantes, France). Eyes from two wild-type and four RPE65 mutant dogs (18–30 months) were used for the study. The animals were euthanized by intravenous injection of pentobarbital sodium (Vétoquinol, Lure, France). The eyes were enucleated, the anterior part and the lens were removed, and the eyecups were fixed for 15–30 minutes in 4% paraformaldehyde and cryoprotected in graded sucrose (10%, 20%, and 30%).

### Immunohistochemistry

**Figure 1:**
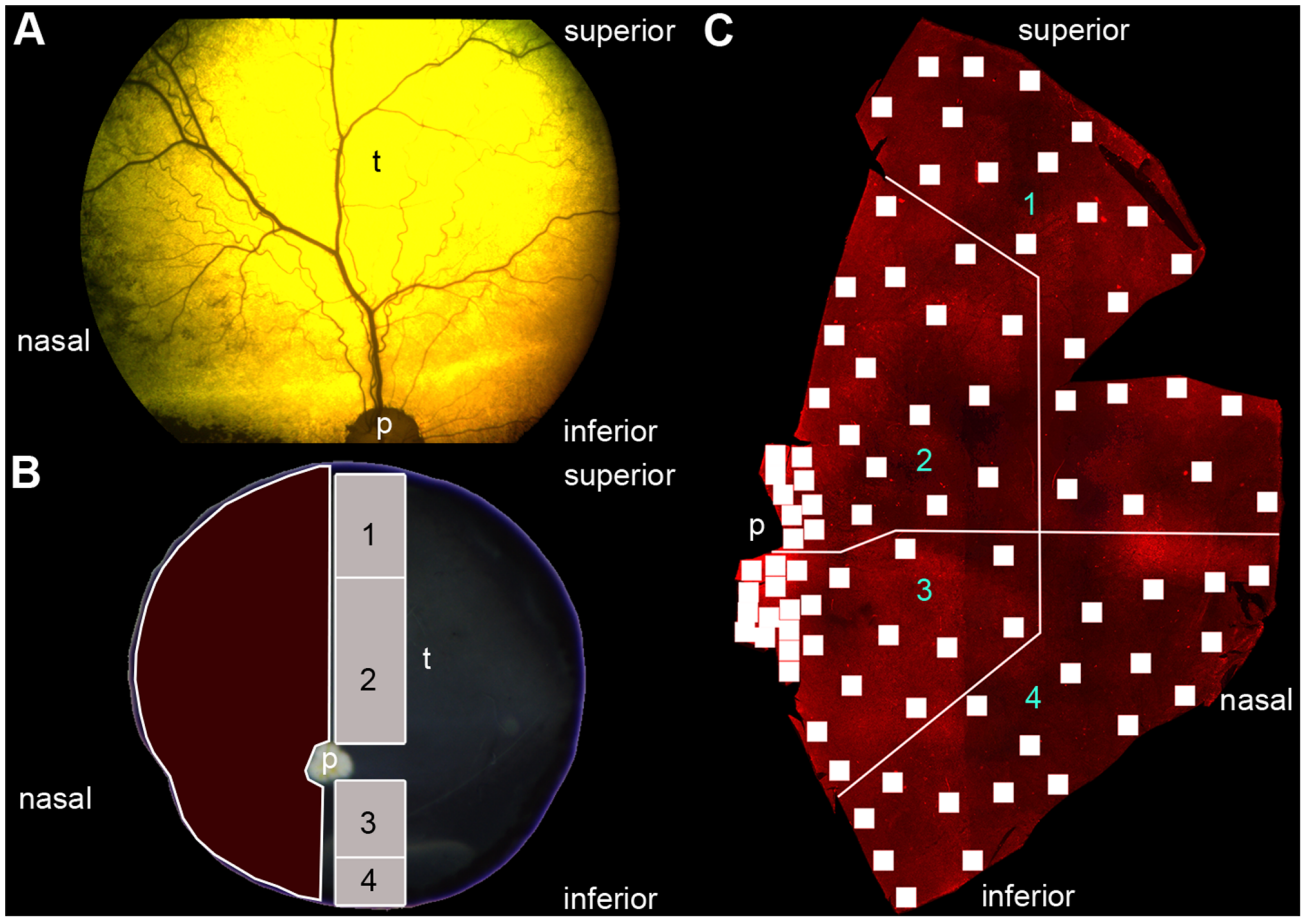
Orientation in the canine eye. (**A**) Funduscopy of a *RPE65^−/−^* dog. (**B**) Extracted eye after euthanasia. The superior part of the eye determines the tapetum lucidum (t). The red marked area corresponds to the retinal flatmount used in the cone opsin expression analysis. The grey marked areas are used for cyrosections used in the bipolar cell sprouting analysis and the numbers correspond to regions shown in Figure 3 und 4. (**C**) Retinal flatmount for cone analysis. Every single quadrat stands for one microscope image (see material and methods). The retina was divided into 4 different regions. The numbers correspond to the regions shown in Figure 2. p = papilla, t = tapetum lucidum.

For cryosections, two different pieces of each retina were excised (Figure 1B). One from the superior ora serrata to the optic nerve head and one from the inferior ora serrata to the optic nerve head. These parts were sectioned vertically at 16 µm on a cryostat embedded in OCT medium (Jung, Nussloch, Germany). Immunocytochemical labeling was done as described previously [37].

For flatmount staining, the nasal part of the retina (Figure 1B) was excised. Retinae were washed several times in PBS at RT and then incubated with a mixture of opsin antibodies diluted in 3% normal donkey serum, 1% bovine serum albumin, and 1% Triton X-100 in PBS for three days at 4°C. After several washing steps in PBS the retinae were incubated with secondary antibodies for 2 h at RT in the same incubation solution as before. Thereafter, retinae were washed in PBS, mounted in Aqua Polymount medium (Polysciences) and stored at 4°C for microscopy. For detailed information about primary antibody see Table 1.

**Table 1:** List of antibodies used in this study.

Antibody	Raised in	Working dilution	Antigen	Source (Cat.No.)
S opsin	Rabbit polyclonal	1∶2000	Recombinant human blue opsin	Chemicon, CA AB 5407)
Calbindin D-28k	Rabbit polyclonal	1∶2000	Recombinant rat calbindin D-28k	Swant,Switzerland (CB-38a)
CtBP2	Mouse monoclonal	1∶10000	Amino acid 361–445 of mouse CtBP2	BDTransduction aboratories, NJ (612044)
L/M opsin	Goat polyclonal	1∶600	Peptide mapping within an extracellular domainof the opsin protein encoded by OPN1LW ofhuman origin	Santa Cruz Biotechnology, CA (sc-22117)
PKC α	Rabbit polyclonal	1∶10000	Synthetic peptide corresponding to amino acids659–672 from the C-terminal variable (V5)region of rat PKCα	Sigma-Aldrich, MO (P4334)

The following secondary antibodies were used: donkey anti-rabbit Alexa 488, donkey anti-mouse Alexa 546 (Life Technology, Darmstadt, Germany), donkey anti-mouse Cy3 and donkey anti-goat Cy3 (Dianova, Hamburg, Germany). All were diluted 1∶500.

### Microscopy

Confocal images were taken using a confocal microscope (AxioMOT with LSM5 Pascal module, Carl Zeiss, Jena, Germany) equipped with Argon and Helium-Neon laser. Images for cone morphology in flatmount preparation were done with a Plan-Apochromat 20x/0.8 (Carl Zeiss, Jena, Germany). The images for sprouting analyses were taken with a Plan-Apochromate 63x/1.4 objective (Carl Zeiss, Jena, Germany). For image adjustment Photoshop CS 5 extended (Adobe Systems, München, Germany) was used.

The images for the cone quantification were captured with a Zeiss Axioplan 2 microscope equipped with epifluorescence. Images were taken with a Plan Neofluoar 2.5x/0.075 objective (Carl Zeiss, Jena, Germany) and afterwards merged in Photoshop CS 5 extended manually to create a map of the retina. The retina map was used to keep the orientation on the preparation for imaging the cones with a Plan Neofluoar 10x/0.30 Ph1 objective (Carl Zeiss, Jena, Germany).

### Data Analysis

#### Sprouting analysis

In each region, six z-stacks (containing 4 successive images with 1 µm distance each) were analyzed. Three images contained double labeling with CtBP2 and PKC, and 3 images contained double labeling with CtBP2 and Calbindin. Quantification of sprouting was done using data of CtBP2 staining of all six images, resulting in data from 24 images per retina. Quantification was done using the Cell Counter Tool (Kurt De Vos, Academic Neurology, University of Sheffield, UK) of Image J (Version 1.47n5, W.S. Rasband, National Institutes of Health, Bethesda, USA). While going through the Z-stack, each clearly visible CtBP2-positive ribbon synapse was counted once. Contrast and brightness of the images were constantly optimized during counting, without altering the original content.

Quantification of ONL thickness was performed using LSM Image Browser software (Version 4.2.0.121, Carl Zeiss Microscopy, Jena, Germany) on the same images where sprouting analysis was done. On each image, the ONL thickness was measured three times at 10 µm distance and the mean values was calculated. Scatter plot with regression curve (first order) was calculated using Sigma Plot.

#### Cone opsin expression analysis

To compare the cone distribution in healthy and mutated retinae, the retina was divided into 4 regions (Figure 1C). For the S-cone distribution, the entire image of the flatmount at each position that was taken with the microscope (0.596 mm^2^) was counted with the manual counting tool of Photoshop CS 5 extended. For the L/M-cones, because of the 10 times higher number of counts per image,only an area of 10% (0.06 mm^2^) was counted. The mean value was calculated for the data of the four affected animals at each position. The mean values of all positions in each region were compared to the healthy control animal with a Mann-Whitney rank sum test using Sigma Plot 12 (Systat Software Inc., San Jose, CA).

**Figure 2:**
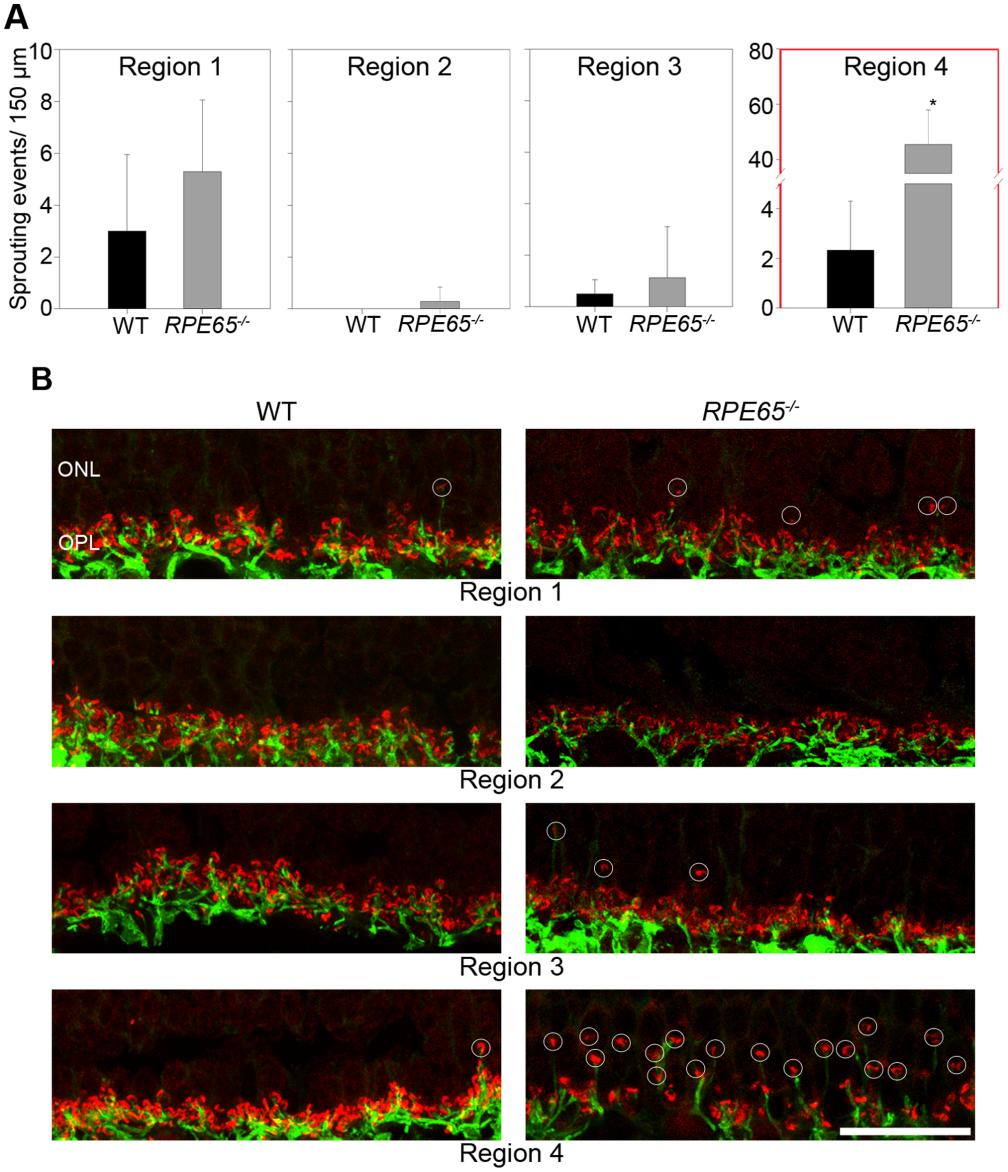
Analysis of sprouting events of rod bipolar cells. (**A**) Bar diagrams of the 4 different analyzed regions (see Figure 1B) compared wild type (n = 12 images) and RPE65^−/−^ (n = 24 images). In regions 1–3 there are no essential differences between wild type and mutated dogs. Region 4 displays a statistical significant discrepancy between wild type and *RPE65^−/−^* dogs (* p<0,001, student t-test). (**B**) High resolution confocal images for the analysis. The images are projections of the 4 considered images of a z-stack. The ribbon synapses are marked with CtBP2 (red) and the rod bipolar cells are marked with PKC α (green). The counted sprouting events are marked with white circles. Wild type (WT), outer nuclear layer (ONL), outer plexiform layer (OPL), scale 20 µm.

To create the heat maps (Figure 2), an interpolation method was used. This method is based on a nearest neighbor approach, using the center coordinates of each sampled region as reference points. Those are sorted according to their distance to any other location in question and the color value at that position is then computed from the mean value of the k nearest reference points, weighted according to their distance to that pixel. The resulting image is produced by applying that procedure to any pixel in the image. Any gray-scale value results from a mapping of a value representing the function-decrease (in percent) to a color-value between 0 and 255. A colored value equivalently results from a mapping to a value between 0 and 1020 which is then interpreted as an RGB-value in a color space comparable to the color temperature measured in degrees Kelvin as it is done in photography and image processing.

## Results

### Experimental Set Up

To examine rod bipolar and horizontal cell sprouting as well as cone opsin expression, parts of four affected retinae and one healthy control retina were used as shown in Figure 1. The tapetal region in the superior retina is shown in Figure 1A. Retinal flatmounts were divided into 4 regions: 1 = superior peripheral, 2 = superior central, 3 = inferior central and 4 = inferior peripheral (Figure 1B,C).

### Sprouting of Rod Synaptic Connections is Dramatically Increased in the Inferior Peripheral Retina (Region 4)

Photoreceptors transmit their chemical signal in the outer plexiform layer (OPL) at the ribbon synapses to the second order neurons (bipolar cells and horizontal cells). The synaptic ribbons of the photoreceptors can be marked with an antibody against C-terminal binding protein 2 (CtBP2) (Figure 2B). The rod terminals (rod spherules) in the OPL normally have one large single ribbon, which appears horseshoe-shaped and is present at high numbers in a rod dominant retina [38]. Rod bipolar cells are ON bipolar cells, which can be labeled with an antibody against PKC α (Figure 2B) [39]. For the horizontal cells, an antibody against the calcium binding protein calbindin was used (Figure S1).

The thickness and the organization of the OPL appear to be unaltered in regions 1, 2, and 3 of affected retinae (Figure 2B). The connections between rods and rod bipolar cells are regular, with some exceptions. In contrast, in the inferior peripheral retina (region 4) a disorganization of the OPL is visible. The ribbon synapses within rod spherules are partially located within the outer nuclear layer (ONL) (Figure 2B), and rod bipolar cell dendrites reach into the ONL. Likewise, horizontal cell processes extend equally into the ONL (Figure S1). This phenomenon is called sprouting and is generally considered to be a marker for non-functional photoreceptors and photoreceptor degeneration [40], [41]. The number of sprouting events in regions 1 and 3 does not differ in affected versus healthy retinae (about 1 to 5 events per 100 µm or a retinal section), indicating normal age-related reorganization. Sprouting was almost not observed in region 2. In region 4 however, a dramatic increase in sprouting events can be observed, reaching 40 to 60 events per 100 µm of a retinal section.

### The Number of Sprouting Events Correlates with ONL Thickness

**Figure 3:**
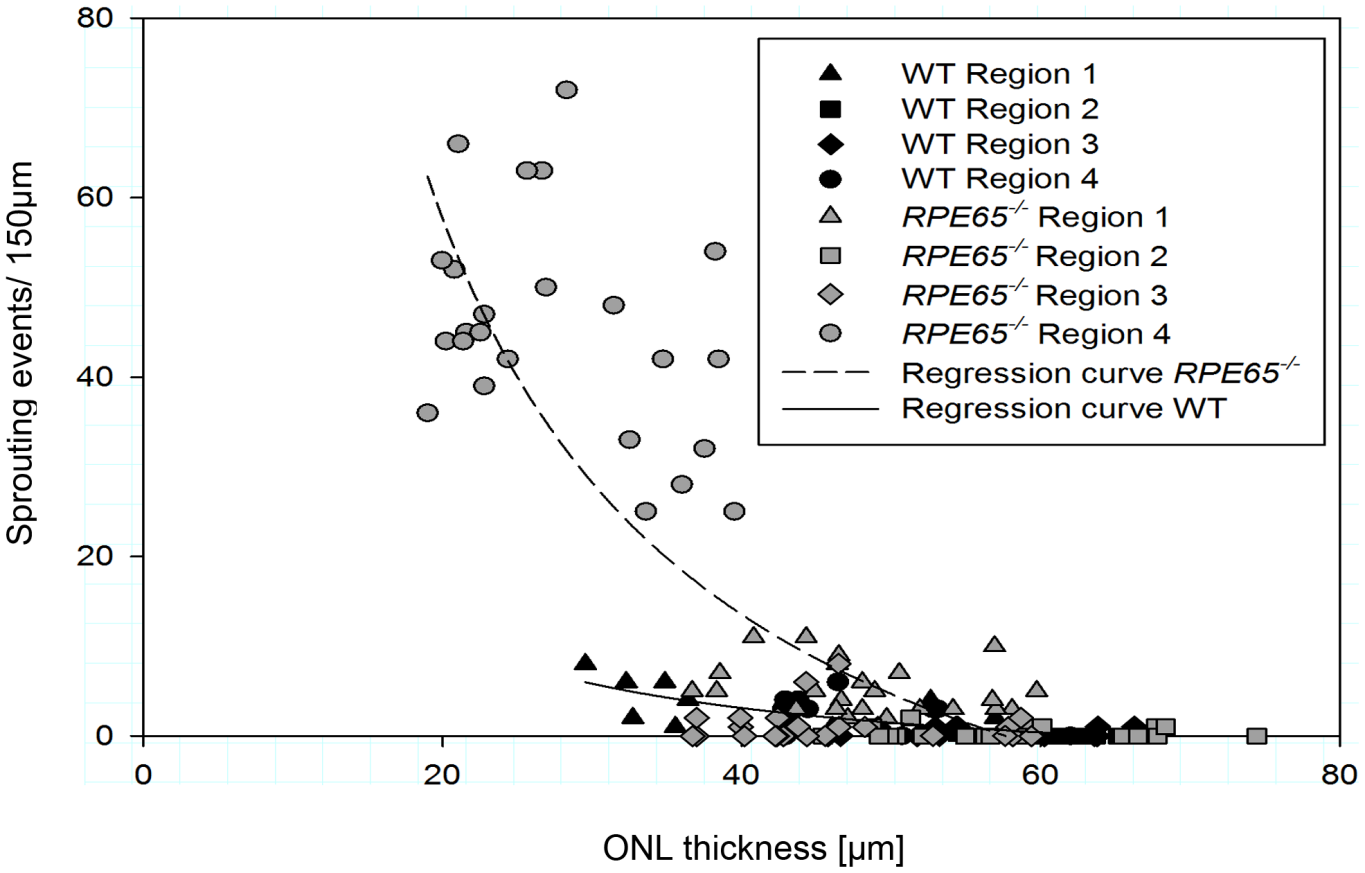
Scatter plot of correlation between sprouting events and ONL thickness. In *RPE65^−/−^* dogs, the highest number of sprouting events as well as the thinnest ONL are present in region 4. There is a strong correlation between sprouting events and ONL thickness (correlation coefficient R = 0.8333 for wild type and R = 0.8374 for *RPE65^−/−^* dogs).

ONL thickness depends on the number of photoreceptor nuclei (mainly rods: 95% of all photoreceptors in dogs) and lies in the range of 30 to 70 µm in the healthy retina, depending on the location being in the periphery or central, respectively (Figure 3). In affected retinae, the thickness of the ONL is not significantly reduced in regions 1 to 3. In contrast, in region 4, the ONL thickness is significantly reduced and correlates with the highest numbers of sprouting events, indicating ongoing photoreceptor degeneration in this area of the retina (Figure 3).

### S-cones are Protected in the Inferior Central Retina (Region 3)

**Figure 4:**
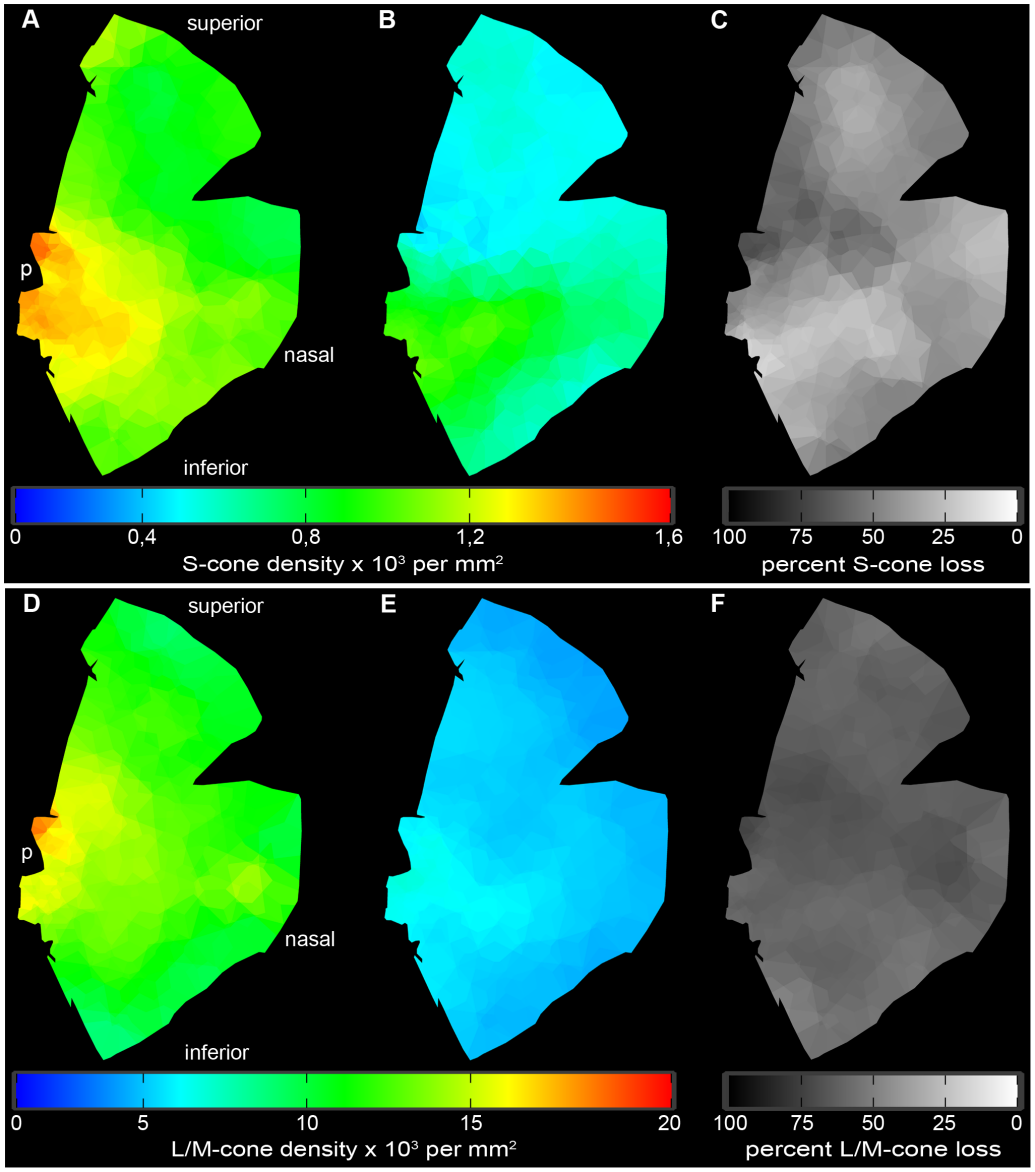
Cone distribution in healthy and affected animals. (**A**) Absolute mean S-cone distribution [10^3^ cones per mm^2^] in wild-type retina (n = 2). (**B**) Absolute mean S-cone distribution [10^3^ cones per mm2] in *RPE65^−/−^* dogs (n = 4). (**C**) Relative mean S-cone loss [%] in the affected animals compared to healthy tissue. (**D**) Absolute mean L/M-cone distribution [10^4^ cones per mm^2^] in wild-type retina (n = 2). (**E**) Absolute mean L/M-cone distribution [10^4^ cones per mm^2^] in *RPE65^−/−^* dogs (n = 4). (**F**) Relative mean LM-cone [%] loss in the affected animals compared to health tissue. p =  papilla.

Following these intriguing results on regional disparities in rod bipolar cell sprouting, we analyzed cone opsin expression in the four different regions of the canine retina. Ninety three areas were analyzed on each retina and the numbers of detectable S- and L/M- cones were visualized on heat maps for absolute and relative loss compared to the healthy retina (Figure 4). The numbers of positive S- and L/M-cones obtained on the healthy control retina were similar to what was observed in a study on cone topography in normal dogs [42].

**Figure 5:**
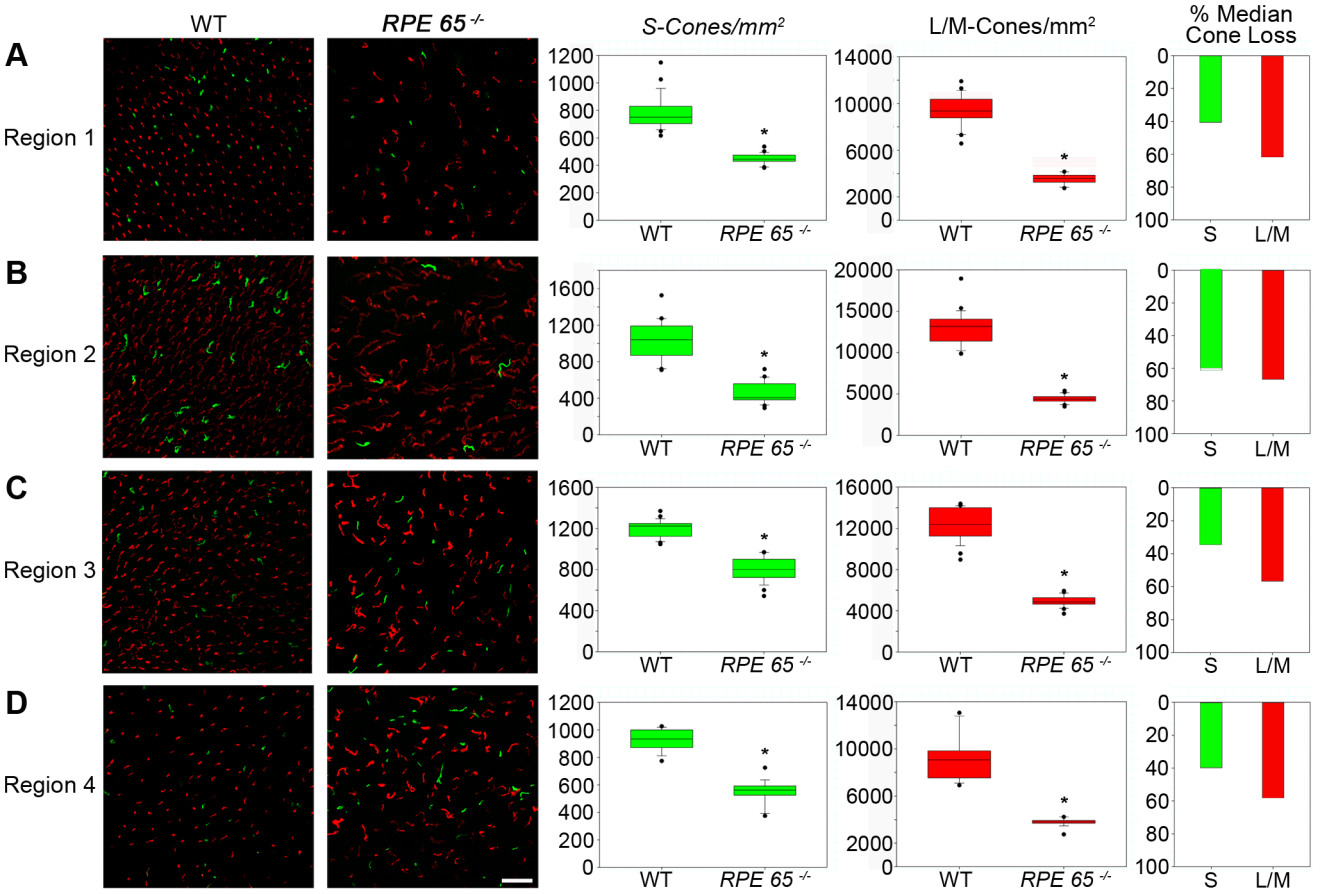
Quantification of cone distribution. (**A–D**) S-cone (green) and L/M-cone (red) distribution and quantification in wild-type and mutated canine *RPE65^−/−^* retinae. Box blots display the respective distributions of S-cones and L/M-cones in the 4 regions defined in Figure 1. Statistical analysis was done by a Mann Whitney rank sum test, significant values are marked with asterisks * p<0.001. Bar diagrams show the percentage loss of both cone types compared healthy and affected animal medians in the analyzed regions. Both types of cones are significantly reduced in all analyzed regions. L/M-cones in *RPE65^−/−^* retinae are not only reduced but look different in shape, thus appearing worm-like. Wild type (WT), scale 25 µm.

The number of S-cones in the wild-type retina is in the range of 1000 cones per mm^2^, with a peak being in the area around the optic disc (1000–1300 per mm^2^), representing the visual streak in canids (Figures 4A and 5B,C). Towards the periphery, the amount of S-cones progressively declines (600–900 per mm^2^) (Figures 4A and 5A,D). The S-cone density in affected animals is significantly reduced in all 4 regions (400–800 per mm^2^), but the reduction is not uniform (Figures 4B,C and 5). The highest S-cone loss with approximately 60% less detectable S-cones takes place in region 2 (Figures 4B,C and 5B). In contrast, S-cone loss is less severe in regions 1, 3, and 4, where pigmented RPE is partially present (region 1) or entirely (regions 3 and 4). In these regions, only about 35% less detectable S cones are present compared to healthy control retinae (Figures 5A, C, and D).

### L/M Cones are not Protected in the Inferior Central Retina (Region 3)

In healthy retinae, similar to S-cone distribution, the highest number of L/M-cones can be found in the two central regions 2 and 3 (10000–15000 per mm^2^) that progressively declines towards the periphery (6000–9000 per mm^2^) (Figures 4D and 5). In affected retinae, the number of detectable L/M-cones is significantly reduced in all 4 regions (Figures 4E and 5). The absolute L/M-cone numbers (3000–5000 per mm^2^) are evenly distributed over the analyzed flatmounts, resulting in a general loss of detectable L/M-cones in all 4 regions at about 60% (58–62%).

## Discussion

To our knowledge, this study analyzes for the first time rod second order neuron sprouting and the cone opsin expression in RPE65 deficient canine retinae with regard to their location within the entire retina. Concerning sprouting of rod second order neuron processes, a phenomenon attributed to ongoing rod degeneration, we observed dramatic activity in the inferior peripheral retina (region 4), while the rest of the retina had significantly less or absent sprouting activity. This sprouting activity correlates with ONL thickness. In addition, we observed a protection of S-cone opsin staining in the inferior central retina (region 3), while L/M cone opsin loss seemed to be independent of the location.

Interestingly, even though the canine retina comprises primarily rod photoreceptors (more than 95%), detailed information about the spatial distribution of degeneration kinetics of these cells in the course of RPE65 deficiency are of limited availability. Initially, the disease in dogs was even considered to be stationary, because no significant degeneration was observed in clinical examinations [18]. However, only the superior part of the retina was examined in clinical settings and information about the disease process in the inferior part was limited to histological images, where the focus was on outer segment organization, which is indeed severely reduced already early in life [20], [21], [25]. In line with this, ONL thinning starts to be detectable in the inferior periphery of the retina, as it was shown on optical coherence tomography (OCT) scans in affected animals at five years of age [35]. In order to study the reaction of rods to RPE65 deficiency, we evaluated the synaptic terminals with second order neurons, i.e. rod bipolar cells and horizontal cells (Figures 2 and S1). We observed intense sprouting of the synaptic connections in the inferior peripheral retina (region 4), which means that already at the age of 2 years, when central retinal thickness values are within the range of healthy tissue, active degenerative processes are ongoing in the inferior periphery. Interestingly, sprouting activity was observed almost exclusively in areas attached to pigmented RPE (region 1, 3, and 4) and similarly in the wild-type retina, albeit to a much lesser extent. Sprouting intensity in the superior periphery (region 1) was much less than in region 4, but still significantly higher than in both central sectors (see Figure 2A).

In a study published by Hernandez et al. (2010) mildly increased dendritic arborisations of bipolar cell into the ONL were observed in affected animals, however information about the localization within the retina and presynaptic elements such as CtBP2 staining is was not given [43].

It is currently unknown, whether the sprouting is triggered by the photoreceptor terminals (presynaptic element) or by second order neurons (postsynaptic element). As observed in our study in regions 1 and 4, sprouting is also detectable in healthy retina, which was previously shown in human and murine eyes [41], [44], representing normal age-related reorganization. However, the level of sprouting that we observed in the inferior periphery (region 4) by far outnumbers this phenomenon.

In current human clinical trials, patients have been treated at ages between 5 and 45 years [27], [28], [30], [34], [45]. As retinal morphology in RPE65 deficiency is already altered in very young patients [9], the treatment will take place in a retina with ongoing neuronal degeneration, no matter at what ages the treatment occurs. Therefore, even photoreceptors that have been successfully rescued by gene therapy may commit to degeneration due to potentially ongoing apoptotic stimuli. A recent publication by Cidecyian et al. (2013) supports this theory, as they observed ongoing ONL thickness loss in successfully treated areas in human patients, but not in dogs treated at a young age [35]. Most animals were treated in the central inferior and superior parts of the retina, where degeneration is retarded and therefore the retina was not in an active state of neurodegeneration. In older animals, ONL protection was not observed. In line with our hypothesis, one dog treated in the inferior retina all the way to the periphery did show functional improvements but not ONL preservation in the periphery. It might therefore be worthwhile considering to study gene therapy in the inferior peripheral part of the canine retina at about 2 years of age, when rod bipolar and horizontal cell sprouting indicate ongoing neurodegeneration in order to model the situation in humans.

An alternative hypothesis would be that only successfully treated photoreceptors stay alive and all untreated cells degenerate. In this case, ONL thickness reduction in treated areas would stop eventually in contrast to untreated areas. Only long-term studies in humans will answer this question.

The second observation made in our study is a less severe reduction of detectable S-cones in the inferior central region (region 3). The same observation was made, albeit to a lesser extent in both peripheral regions (region 1 and 4).(regions 1 and 4). These three regions are those, which are attached to pigmented RPE. This observation seems to be contradictory to what we observed concerning the sprouting events in the rod pathway, which were highest in retinae attached to pigmented RPE. To make things even more complicated, L/M cone opsin reduction seems to be independent of the location within the retina. One explanation would be that there is a protective agent or mechanism that prevents S-cones from dying. This S-cone protective effect has not yet been reported in the literature, partly because this study is the first one to address this question in the entire canine retina. Mowat et al. (2013) compared the photoreceptor density of affected RPE65 dogs at 3 months and 6.5 years of age, and found a reduction of cone and rod labeling between young and old animals [46]. They observed a higher loss of S-cones compared to L/M-cones over time. Different regions within the superior part of the retina were analyzed and cone loss was higher in the center compared to the periphery. However, they did not compare inferior versus superior parts of the retina, as we did in our study, nor did the authors compare the data with those of unaffected animals. In a second study, Hernández et al. (2010) found no changes in the total number of cones in mutated retinae at 17 months of age [43]. This discrepancy compared to our study is due to the fact that the authors stained the cone cell bodies with human cone arrestin (hCAR), while we detected the cone opsins inside the cells. Lack of a reduction of hCAR staining in one study and reduced opsin staining in our study in animals at about the same age indicate that the number of cone cell bodies may be consistent while the function of the cells as indicated by opsin expression is already reduced. In contrast to our observations, a recent study in dogs affected with achromatopsia due to mutations in the CNGB3 gene did show the highest protection of S cone opsin expression in the superior central area attached to non-pigmented RPE (corresponding to our region 2) [47]. However, because the pathology in achromatopsia is different, reasons for this difference between the studies may be manifold.

A correlation of S cone protection and pigmentation seems to be possible since the pigment in the RPE cell layer has specific functions such as light absorption and antioxidative activity, both rendering it light protective [48]. This light protective nature of pigment granules could well be the reason of why the S-cone loss is less severe in regions attached to pigmented RPE. On the other hand, the uniform loss of L/M-cones seems to contradict this theory. One reason for this discrepancy could be light induced stress of photoreceptors. Light stress in canine retinae is not evenly distributed, as the tapetum lucidum reflects the light in the central superior region (region 2), enabling increased photon capture in this area of the retina [36]. However, little is known about the characteristic of the light reflected by the tapetal cells in dogs and therefore, it remains unknown whether local differences of reflection are causative for the uneven S-cone photoreceptor loss.

One possible explanation that is independent of pigmentation are differences in the intracellular stress response in the two cone photoreceptor subtypes, which was demonstrated by Zhang et al (2011) *in vitro* after transfection of human and murine cone opsins into COS7 cells and also *in vivo* in Lrat^−/−^ mice [49]. Under 11-*cis* retinal deprivation, L/M- and S-cone opsins have different aggregation properties causing accumulation of opsin in the endoplasmic reticulum. This causes activation of the unfolded protein response, which may induce apoptosis. It was shown that, compared to L/M-opsin, more S-opsin accumulated in inner segments and OPL and was co-localized with ubiquitin. This observation could also explain the different kinetics of cone opsin loss observed in our study.

In this study, we demonstrate that in RPE65 deficient dogs rod second order neuron sprouting is heavily increased in the inferior periphery at two years of age and generally increased in areas attached to pigmented RPE. In contrast, we show that S cone opsin expression is not uniformly reduced, as pigmented RPE seems to preserve S-cone opsin but not L/M-cone opsin expression. As the medical field deals with the results of the RPE65 gene therapy, the data obtained in this study add further information on the pathology in the most relevant large animal model and should be considered when designing further preclinical studies.

## Supporting Information

Figure S1 **Analysis of sprouting events of horizontal cells.** Confocal images are projections of 4 considered images of a z-stack. The ribbon synapses are marked with CtBP2 (red) and the horizontal cells are marked with Calbindin (green). The detected sprouting events are highlighted with white circles. Wild type (WT), outer nuclear layer (ONL), outer plexiform layer (OPL), scale 20 µm.
(TIF)
