# Supplementary figures and images for: Immuno-Histochemical Analysis of Rod and Cone Reaction to RPE65 Deficiency in the Inferior and Superior Canine Retina
Source: PLoS One. 2014 Jan 21;9(1):e86304. doi: 10.1371/journal.pone.0086304 (PMC3897682; doi:10.1371/journal.pone.0086304)

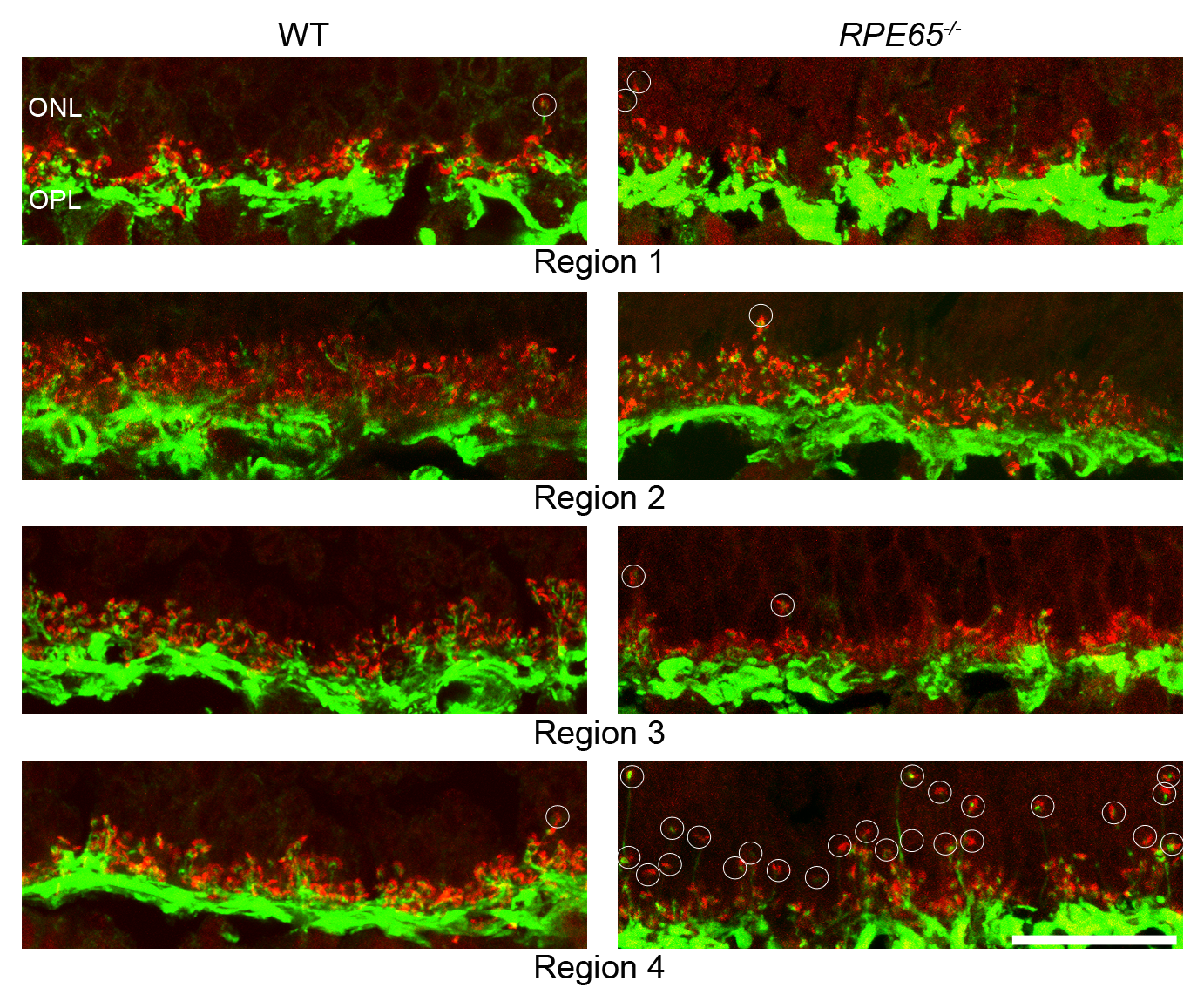

Supplement: Figure S1 — Analysis of sprouting events of horizontal cells. Confocal images are projections of 4 considered images of a z-stack. The ribbon synapses are marked with CtBP2 (red) and the horizontal cells are marked with Calbindin (green). The detected sprouting events are highlighted with white circles. Wild type (WT), outer nuclear layer (ONL), outer plexiform layer (OPL), scale 20 µm. (TIF) [file pone.0086304.s001.tif]
